# Supplementary material for: Fully automated multicolour structured illumination module for super-resolution microscopy with two excitation colours
Source: Commun Eng. 2025 Mar 10;4:42. doi: 10.1038/s44172-025-00365-x (PMC11894046; doi:10.1038/s44172-025-00365-x)
Supplement: Supplementary file 2 — Supplementary PDF [file 44172_2025_365_MOESM2_ESM.pdf]

Supplemental Information for Fully-Automated Multicolour Structured Illumination Module for Super-resolution Microscopy with two Excitation Colours

Haoran Wang *et al.*

S1. SAMPLE PREPARATION

HeLa cells were cultured in Dulbecco's Modified Eagle Medium (DMEM) supplemented with 10 % Fetal bovine serum (FBS) and 1 % penicillin/streptomycin solution (P/S) and maintained in a 37°C/5 % CO2 incubator. Cells were harvested around 90% confluency with TrypLE express 1x (12604013, ThermoFisher Scientific, Germany) and transferred into ibidi 4 well slide (µ-Slide 4 well, ibidi GmbH, Germany), the sample slide is cultured again for one day to make sure cells adhere to the glass bottom.

For live cell imaging, cells were stained with MitoTracker Green (Invitrogen MitoTracker Green, ThermoFisher Scientific, Germany) and imaged using 488 nm laser. To prepare the staining solution, 1 µL MitoTracker Green stock solution was diluted into 1 mL cell culture medium in order to produce staining solution with concentration of 1 µM. When the cells achieve 70%-90% confluency, remove the old culture medium and wash the sample with PBS three times, then add the staining medium to the slide and culture the sample in the incubator for 1 h before imaging.

When the HeLa cells in Ibidi slide multiply up to 70%-90% confluency, the sample was fixed with 4 % Paraformaldehyde solution (PFA) for 15 min at room temperature. After fixation, the sample was washed with PBS three times in order to remove the PFA solution totally. 0.1 % Triton X-100 diluted in PBS was used to permeabilize the cell membrane for 2 min. 2 µL Alexa Flour 488 Phalloidin stock solution was diluted with 400 µL PBS as staining solution for each well. The sample was stained with staining solution for 50 min at room temperature and later stored in PBS solution.

S2. LINEPLOT OF ARGOLIGHT CALIBRATION SAMPLE

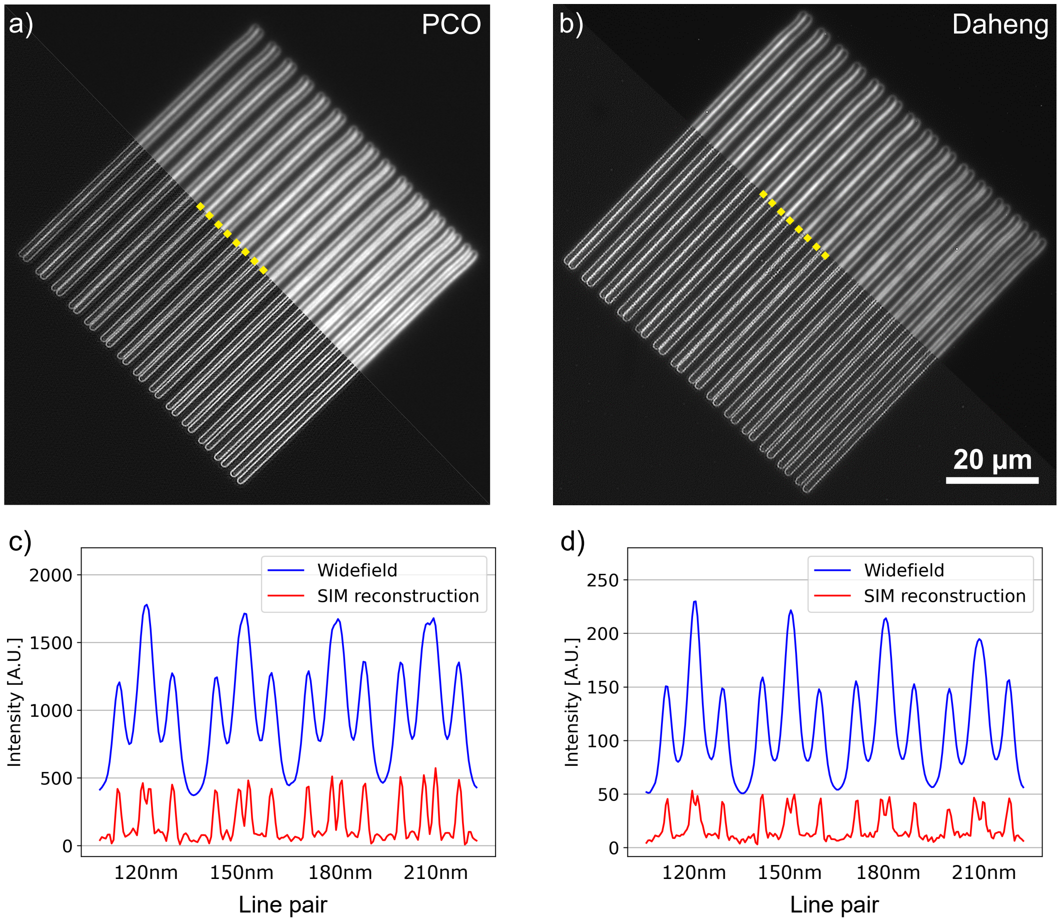

FIG. S1. **Characterization of the system properties:** Resolution characterization of the system with different cameras including the intermediate magnification lens. The data show the line pair on the Argolight structured illumination microscopy (SIM) calibration slide excited with a 488 nm laser. Wide-field and SIM results with a) the PCO Edge 4.2 camera and b) the Daheng MER2-230-168U3M camera. c) and d) show the line plot of the line pair between 120 nm and 210 nm. The line plot data is averaged over 5 pixels.

S3. IMAGING WITH INDUSTRIAL CAMERA

For a cost-effective solution, an industrial grade CMOS camera (MER2-230-168U3M, Daheng, China) was tested on the setup. The camera has a CMOS chip IMX174 with pixel pitch of 5.86 µm. The widefield image with SIM reconstruction of a FluoCells Nr. 1 slide is shown in Fig. S2.

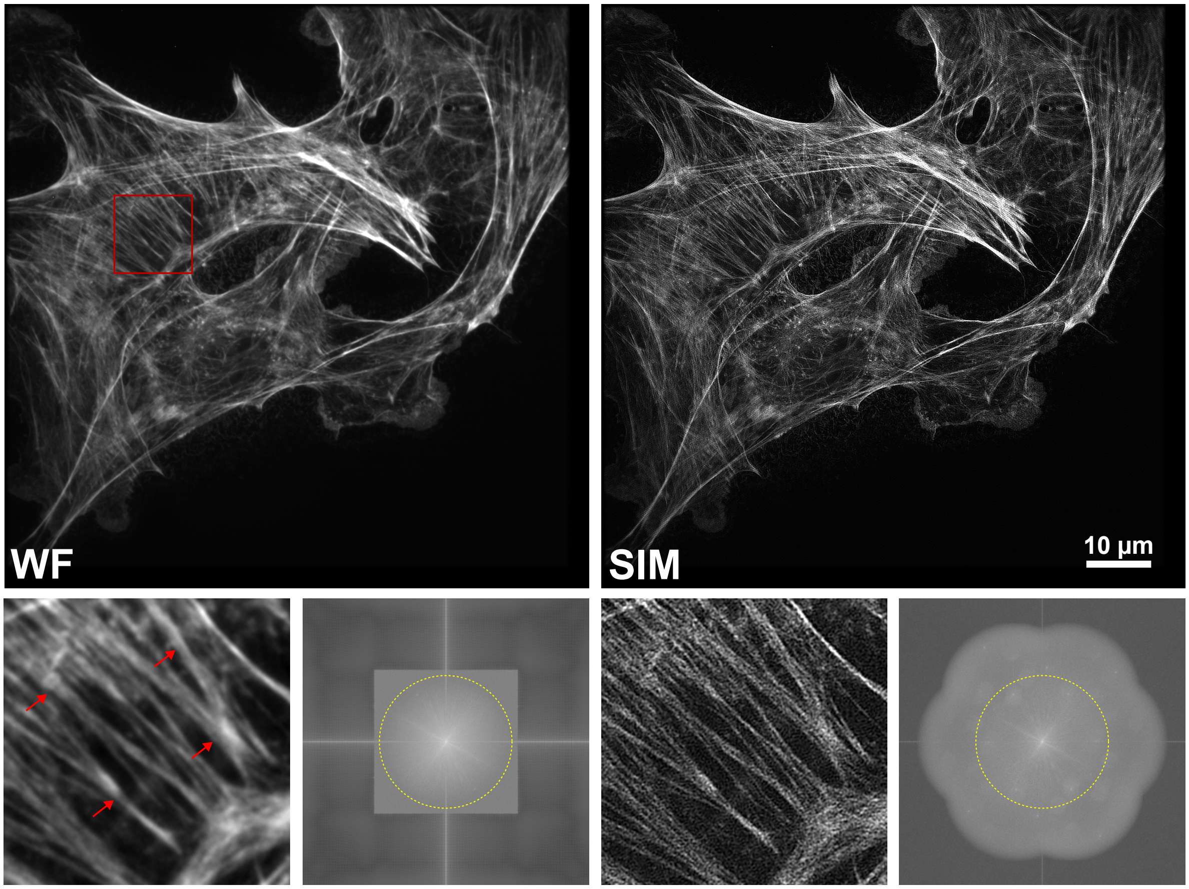

FIG. S2. **Comparing industry-grade and scientific-grade cameras for super-resolution imaging:** BPAE cells from a FluoCells Nr. 1 slide were imaged using the Daheng camera. We compare the widefield image (upper left) and structured illumination microscopy (SIM) reconstruction (upper right), SIM reconstruction gives better resolution compared to the widefield. To illustrate the enhanced SIM resolution we display a region of interest (lower left and third from left). To illustrate the increased frequency support in Fourier space, we also display the Fourier transforms of the widefield image (lower, second from left) and SIM reconstruction (lower, right).

S4. SIM PATTERN GENERATIONS

The DMD patterns used to project the SIM illumination were generated using the fastSIM grating search algorithm[1] implemented in fairSIM[2]. The pattern search algorithm can tune the illumination pattern based on different hardware modalities, e.g. objective NA and system magnification. For different experiments, we used SIM patterns designed to provide resolution enhancements of 1.55, 1.65, or 1.75 times. The DMD pattern parameters are

provided in table S1. In order to capture the pattern information with smaller grating constant, an in-built 1.5× intermediate magnification lens (IML) in the Nikon Ti2-A Eclipse was employed in the imaging path. In this work, we used the 1.55 × patterns for the dual color data presented in the main manuscript. We used 1.65× and 1.75× patterns for 488 nm excitation to compare the results in Fig. S3.

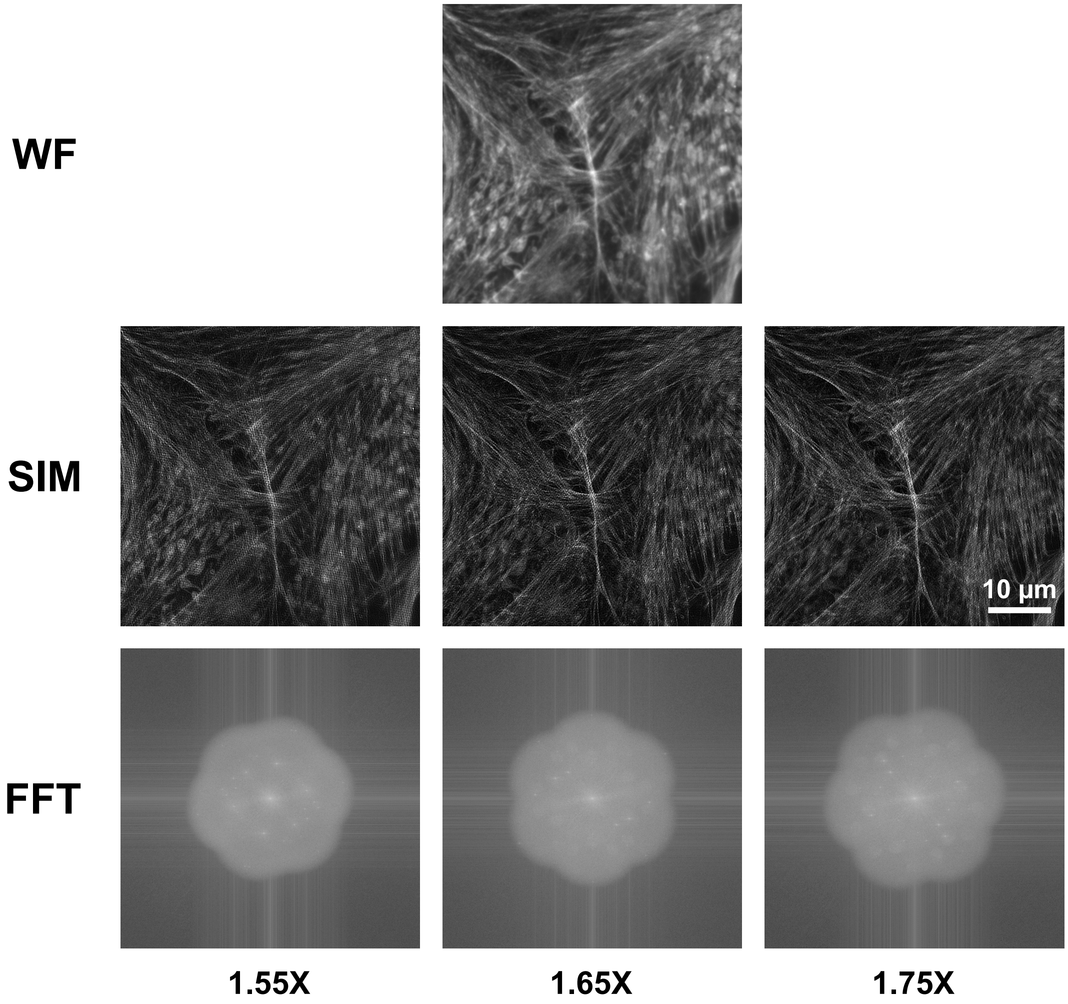

FIG. S3. **Demonstration of resolution improvements for different patterns:** Different patterns were used in the experiment to see the difference between varied grating constant for the resolution enhancement. The sample shown here is stained BPAE cells on FluoCells Nr. 1 and excited at 488 nm. The results show with finer grating applied to the sample, the resolution of the image is slightly increased, which is also proved on the Fourier transformation of the data with more higher frequency information. Data were captured with PCO Edge 4.2 camera.

Enhancement	excitation (nm)	$\mathbf{V}_a$	$\mathbf{V}_b$	d (mirrors)	period (nm)
		$(9, 10)$	$(2, -8)$	6.838	
1.55×	488	$(9, -28)$	$(2, -29)$	6.97	307 nm
		$(19, -4)$	$(4, 6)$	6.695	
		$(17, 19)$	$(5, -8)$	9.061	
1.55×	635	$(8, -25)$	$(4, 18)$	9.296	408 nm
		$(14, -3)$	$(5, 8)$	8.87	
		$(0, 9)$	$(5, 4)$	5.925	
1.65×	488	$(0, -30)$	$(6, -30)$	6	267 nm
		$(2, 5)$	$(-3, 2)$	5.831	
		$(3, 4)$	$(2, -6)$	5.2	
1.75×	488	$(3, -7)$	$(2, -18)$	5.252	234 nm
		$(25, -3)$	$(2, 5)$	5.203	

TABLE S1. DMD SIM pattern parameters for the various patterns considered in this work.

S5. 3D DATA WITH CROSS SECTION

A stepper motor was attached to the fine focus knob of the microscope body and it gives the setup the possibility to take 3D data along Z-axis. A 3D view of a data is shown in Fig. S4. A cross section view of the position pointed with dotted line is plotted below. In the XZ-plane plot, the SIM reconstruction provides sectioning ability and removes some background signal compare to widefield.

S6. EFFECT OF POLARIZATION ON PATTERN CONTRAST

In coherent SIM, the control of polarization directions critically influences the intensity contrast of the illumination patterns. Perfect interference occurs when the light is polarized orthogonal to the plane formed by the beam propagation direction and the optical axis, while for light polarized parallel to this plane the contrast m is reduced to $\cos(2\theta)$, where θ is the angle between the incident SIM beams and the optical axis. Note that the contrast m is negative for $\theta > \pi/4$, just indicating a flip of the pattern phase. Averaging over various input directions, such as with circularly or unpolarized light, the modulation contrast becomes

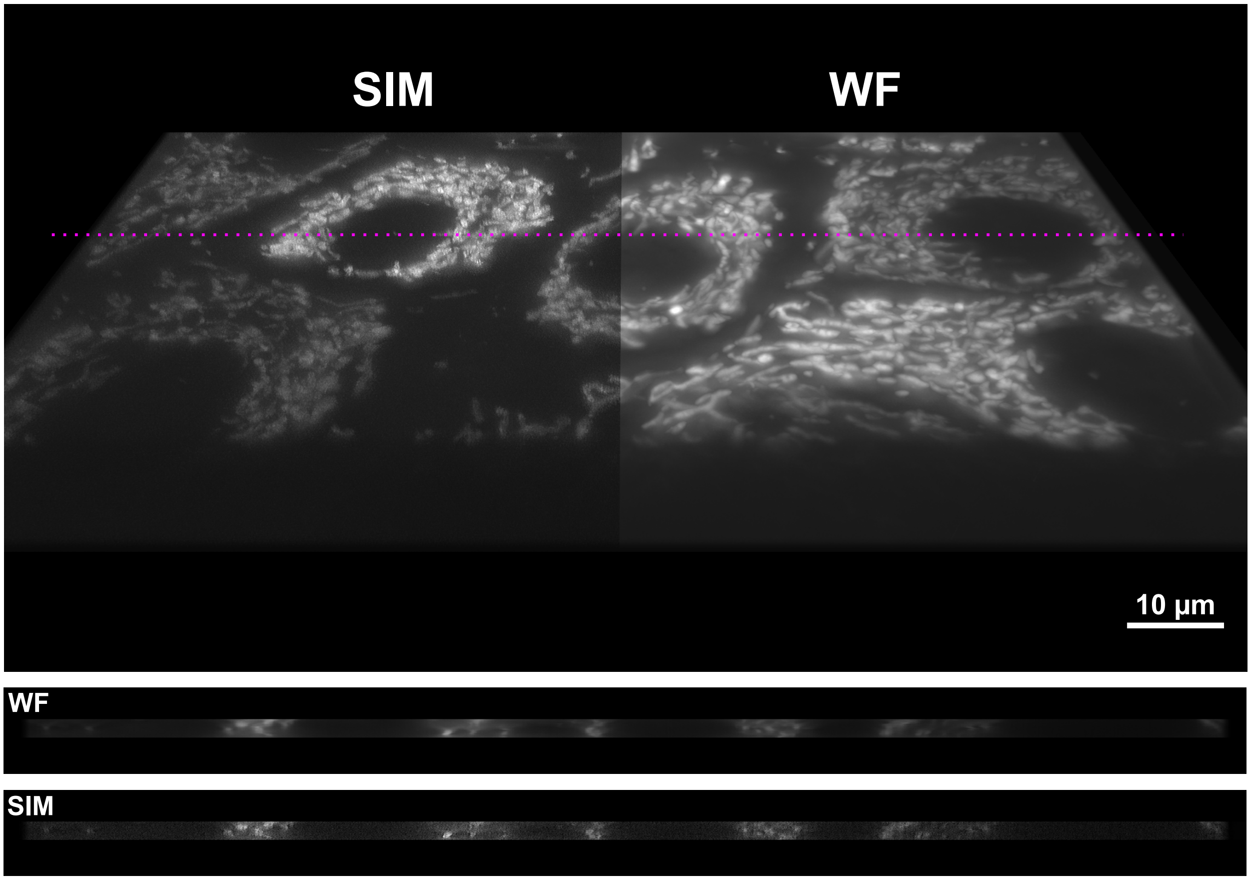

FIG. S4. **Volumetric super-resolution imaging results of HeLa cells:** A 3D view of HeLa cell labeled with MitoTracker Green. The cross section shows an resolution enhancement on Z direction. The data was captured with PCO camera with 300 nm spacing over 9 μm.

$0.5\,(1+\cos(2\theta))^3$. Although linearly polarized light can provide excellent interference at one SIM angle, it may perform poorly at others. If the polarization cannot be controlled separately for different SIM angles, circularly polarized light is more advantageous, as 'half' of it interferes perfectly.

In reality, the light exiting the single-mode fiber coupled lasers is neither linear nor circularly polarized leading to a maximum achievable pattern contrast of $\frac{I_{\max}-I_{\min}}{I_{\max}+I_{\min}} \sim (0.6, 0.3, 0.2)$ for the three different rotational angles for SIM pattern period 310 nm, NA = 1.4, and the 488 nm excitation. These values were calibrated with a sparse bead sample (TetraSpeck microspheres, 0.1 μm, Thermo Fisher, MA, USA). In our experiments, we manually wiggled the fibers to maximise pattern contrast in the sample plane. In reality, the light exiting the single-mode fiber coupled lasers is neither linear nor circularly polarized leading to a

maximum achievable pattern contrast of $\frac{I_{\max}-I_{\min}}{I_{\max}+I_{\min}} \sim (0.6, 0.3, 0.2)$ for the three different rotational angles for SIM pattern period 310 nm, NA = 1.4, and the 488 nm excitation. These values were calibrated with a sparse bead sample (TetraSpeck microspheres, 0.1 µm, Thermo Fisher, MA, USA). This contrast, although reduced, still surpasses that achievable with incoherent SIM, thereby enhancing resolution reconstruction. In our experiments, we manually optimized the maximum available pattern contrast in the sample plane by twisting the fibers to select the polarization state.

.

S7. DMD MODEL

A. Blaze condition optimization

Here we discuss the solution to the DMD diffraction optimization problem described in the main text. We have 8 unknowns, including the Lagrange multipliers, and 8 equations. However, since the optimization problem only depends on the vector difference $\hat{\mathbf{b}} - \hat{\mathbf{a}}$, we will find one of these equations is redundant.

To evaluate the derivatives of the cost function, we use the basis change expressions,

$$b_1 - a_1 = R_{11}(b_x - a_x) + R_{21}(b_y - a_y) + R_{31}(b_z - a_z) \tag{S1}$$

$$b_2 - a_2 = R_{12}(b_x - a_x) + R_{22}(b_y - a_y) + R_{32}(b_z - a_z), \tag{S2}$$

Then equation 8 becomes

$$\alpha + 2(b_1 - a_1)\left[R_{11} - R_{31}\frac{b_x}{b_z}\right] + 2(b_2 - a_2)\left[R_{12} - R_{32}\frac{b_x}{b_z}\right] = 0 \tag{S3}$$

$$-\alpha + 2(b_1 - a_1)\left[-R_{11} + R_{31}\frac{a_x}{a_z}\right] + 2(b_2 - a_2)\left[-R_{12} + R_{32}\frac{a_x}{a_z}\right] = 0 \tag{S4}$$

$$\beta + 2(b_1 - a_1)\left[R_{21} - R_{31}\frac{b_y}{b_z}\right] + 2(b_2 - a_2)\left[R_{22} - R_{32}\frac{b_x}{b_z}\right] = 0 \tag{S5}$$

$$-\beta + 2(b_1 - a_1)\left[-R_{21} + R_{31}\frac{a_y}{a_z}\right] + 2(b_2 - a_2)\left[-R_{22} + R_{32}\frac{a_x}{a_z}\right] = 0. \tag{S6}$$

Adding eqs. S3 and S4 and separately S5 and S6 gives

$$2[(b_1 - a_1)R_{31} + (b_2 - a_2)R_{32}]\left(\frac{b_x}{b_z} - \frac{a_x}{a_z}\right) = 0 \tag{S7}$$

$$2[(b_1 - a_1)R_{31} + (b_2 - a_2)R_{32}]\left(\frac{b_y}{b_z} - \frac{a_y}{a_z}\right) = 0. \tag{S8}$$

This leaves two possibilities (i) both terms in round brackets are zero, implying $\hat{\mathbf{b}} = -\hat{\mathbf{a}}$ and the solution is unique or (ii) the term in square brackets is zero, and there is a one-parameter family of solutions.

In case (ii) we have

$$2[(b_1 - a_1)R_{31} + (b_2 - a_2)R_{32}] = 0. \tag{S9}$$

Substituting eqs. S1 and S2 into eq. S9, we find,

$$b_z - a_z = -\frac{\lambda}{d}\frac{n_x(R_{31}R_{11} + R_{32}R_{12}) + n_y(R_{31}R_{21} + R_{32}R_{22})}{R_{31}^2 + R_{32}^2}. \tag{S10}$$

Combining this result with the diffraction condition fixes the value of $\hat{\mathbf{b}} - \hat{\mathbf{a}}$. In principle, we also know the values of the Lagrange multipliers by reinserting eq. S10 into eqs. S3–S6.

We have three parameters left to fix, which are the components of either $\hat{\mathbf{a}}$ or $\hat{\mathbf{b}}$. However, since the cost function only depends on the difference vector, this will not affect its value. Therefore, we have full freedom to choose one unit vector component, say a_x, and we will still have a solution to our equations. That is, our system of equations is actually under-determined because two of the Lagrange multiplier equations were identical.

Therefore, it remains only to express a_y in terms of a_x and $\hat{\mathbf{b}} - \hat{\mathbf{a}}$. One convenient approach is to notice that combining eqs. 3, 4, and S10 also fixes $\hat{\mathbf{b}} \cdot \hat{\mathbf{a}}$ in terms of the DMD geometric parameters and the diffraction order

$$\begin{aligned} \hat{\mathbf{b}} \cdot \hat{\mathbf{a}} &= \frac{1}{2}\left[2 - (\hat{\mathbf{b}} - \hat{\mathbf{a}}) \cdot (\hat{\mathbf{b}} - \hat{\mathbf{a}})\right] \\ &= 1 - \frac{1}{2}\left(\frac{\lambda}{d}\right)^2 \left(n_x^2 + n_y^2 + \left[\frac{n_x(R_{31}R_{11} + R_{32}R_{12}) + n_y(R_{31}R_{21} + R_{32}R_{22})}{R_{31}^2 + R_{32}^2}\right]^2\right) \end{aligned} \tag{S11}$$

We now derive a quadratic equation for a_y in terms of a_x, where all other vector parameters are expressed in terms of a_x, the chosen diffraction order, and the DMD geometric parameters. To do this, we rewrite the dot product in terms of components, eliminate the z-components using eq. 1 and eq. 2, and square both sides

$$b_x a_x + b_y a_y - \hat{\mathbf{b}} \cdot \hat{\mathbf{a}} = -b_z a_z \tag{S12}$$

$$\left[b_x a_x + b_y a_y - \hat{\mathbf{b}} \cdot \hat{\mathbf{a}}\right]^2 = \left(1 - a_x^2 - a_y^2\right)\left(1 - b_x^2 - b_y^2\right). \tag{S13}$$

Finally, we rewrite eq. S13 in the form

$$\begin{aligned} &a_y^2 \left[2\left(b_x a_x - \hat{\mathbf{b}}\cdot\hat{\mathbf{a}}\right) + \left(1-b_x^2\right) + \left(1-a_x^2\right)\right] + \\ &a_y \left[2n_y\frac{\lambda}{d}\left(b_x a_x - \hat{\mathbf{b}}\cdot\hat{\mathbf{a}} + 1 - a_x^2\right)\right] + \\ &\left[\left(b_x a_x - \hat{\mathbf{b}}\cdot\hat{\mathbf{a}}\right)^2 - \left(1-a_x^2\right)\left(1-b_x^2\right) + \left(n_y\frac{\lambda}{d}\right)^2\left(1-a_x^2\right)\right] = 0, \end{aligned} \tag{S14}$$

where we have explicitly eliminated b_y by inserting eq. 4 and implicitly eliminated b_x and $\hat{\mathbf{b}}\cdot\hat{\mathbf{a}}$ using eqs. 3 and S11 respectively. This quadratic equation can now be solved for a_y as a function of a_x. Since eq. S14 was obtained by squaring eq. S12, we must check that the resulting solutions also satisfy eq. S12. Those that do are the final solutions to our optimization problem and recover the exact solutions to the combined diffraction and blaze conditions, if they exist.

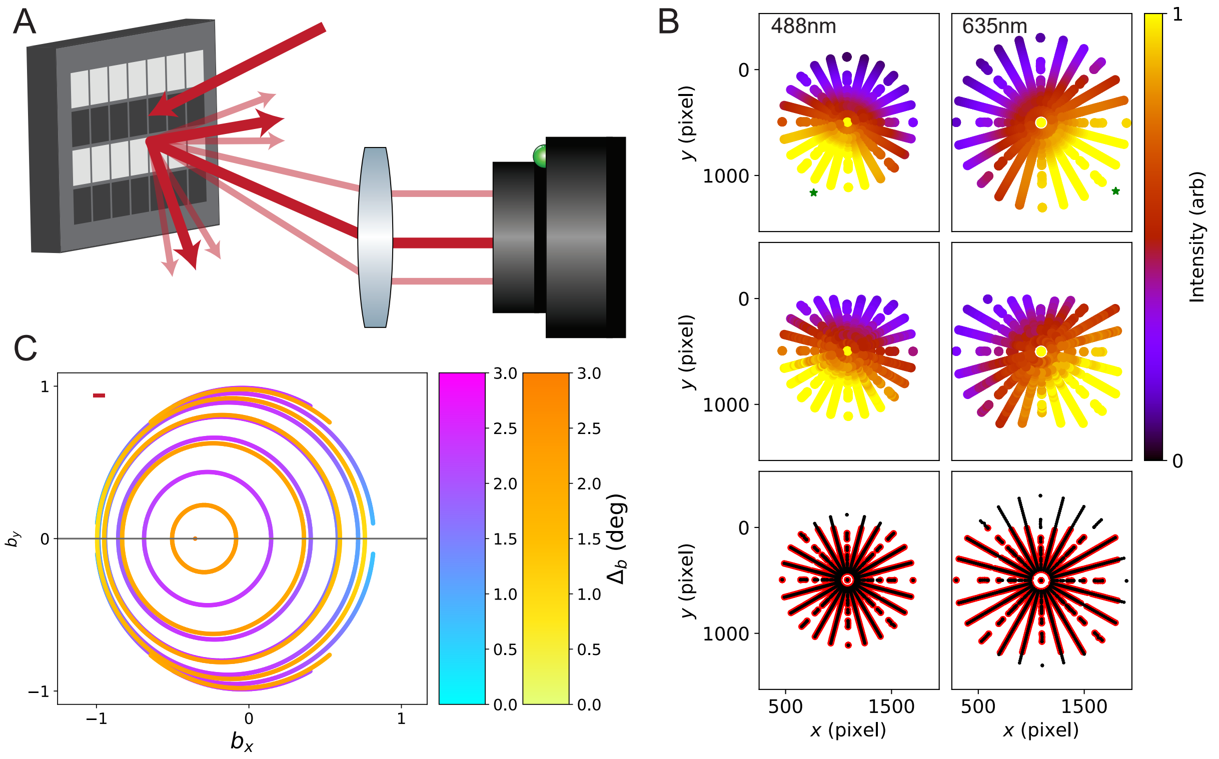

FIG. S5. **Simualatting the effect of different diffraction angles on the digital mirror device (DMD):** A). Coherent light incident on the DMD pattern is diffracted into many orders, and the intensity and position of some of these orders are measured on a camera placed after a lens in a $2f$-configuration. B). We compare the predictions of our DMD diffraction model (top row) to the experimental measurements (second row) after applying an optimization procedure to determine the most likely beam geometry and DMD parameters. The predicted centers of the blaze envelope are shown (green stars) C). We recompute the optimally blazed DMD solutions using the recovered parameters, and find that due to deviations between the nominal and actual mirror rotation, the achievable blaze angle violation is a factor of ~ 3 worse than expected. We consider diffraction orders $(-n, 0)$ for $n = 1, ..., 6$. For 488 nm, the innermost closed curve corresponds to the $(-6, 0)$ order. For 635 nm, the $(-6, 0)$ order solution is a single point, and the first closed curve corresponds to $(-5, 0)$.

B. Pixel Rotation Matrices

We can express any rotation matrix as a rotation through angle γ about axis $\hat{\mathbf{m}} = (m_x, m_y, m_z)$. In this case, the rotation matrix is given by

$$R(\hat{\mathbf{m}},\gamma) = \begin{pmatrix} m_x^2(1-\cos\gamma)+\cos\gamma & m_xm_y(1-\cos\gamma)-m_z\sin\gamma & m_xm_z(1-\cos\gamma)+m_y\sin\gamma \\ m_xm_y(1-\cos\gamma)+m_z\sin\gamma & m_y^2(1-\cos\gamma)+\cos\gamma & m_ym_z(1-\cos\gamma)-m_x\sin\gamma \\ m_xm_z(1-\cos\gamma)-m_y\sin\gamma & m_ym_z(1-\cos\gamma)+m_x\sin\gamma & m_z^2(1-\cos\gamma)+\cos\gamma \end{pmatrix}.$$

C. Corner Illumination Pixels

For CIP's, the rotation matrix parameters are

$$R^{\pm} = R\left(\frac{\hat{\mathbf{e}}_x + \hat{\mathbf{e}}_y}{\sqrt{2}}, \gamma_{\pm}\right) \tag{S15}$$

with $\gamma_{\pm} = \pm 12°$. Since $R_{13} = -R_{23}$, we only have exact solutions for $n_x = -n_y = n$, and in this case eq. 6 implies

$$\begin{aligned} a_3 &= \frac{\lambda}{d}\frac{n}{\sqrt{2}\sin\gamma} \\ a_1 &\in \Big[-(1-|a_3|)\,, 1-|a_3|\Big] \\ a_2 &= \pm\sqrt{1-a_3^2-a_1^2}. \end{aligned}$$

D. Tilt and roll pixels

The TRP's use a mechanism that tilts the mirror first by 12° degrees along one diagonal, then rolls it ±12° along the other diagonal to reach the + and − states, respectively. The + position is tilted approximately 17° along the y-direction, while the − position is tilted approximately −17° degrees along the x-direction[4]. Rewriting each composite rotation along a single axis,

$$R^{+} = R\left(\frac{\hat{\mathbf{e}}_x + \hat{\mathbf{e}}_y}{\sqrt{2}}, 12°\right) R\left(\frac{\hat{\mathbf{e}}_x - \hat{\mathbf{e}}_y}{\sqrt{2}}, 12°\right) \approx R(0.997\hat{\mathbf{e}}_x - 0.074\hat{\mathbf{e}}_z, 16.96°) \tag{S16}$$

$$R^{-} = R\left(\frac{\hat{\mathbf{e}}_x + \hat{\mathbf{e}}_y}{\sqrt{2}}, -12°\right) R\left(\frac{\hat{\mathbf{e}}_x - \hat{\mathbf{e}}_y}{\sqrt{2}}, 12°\right) \approx R(0.997\hat{\mathbf{e}}_y - 0.074\hat{\mathbf{e}}_z, -16.96°). \tag{S17}$$

Parameter	Expected Value	Fit Value	Comments
Blue diffraction order	(-6, 0)		
Red diffraction order	(-5, 0)		
DMD pitch (µm)	5.4	5.4	Constrained to $\pm 0.001\,\mu\text{m}$ of guess
λ_1 (nm)	488(1)	488.0(2)	Constrained to [487,489]
$\theta_{x,1}$ (°)	37	38.2(3)	$\hat{\mathbf{a}}_1 = (0.618, -0.003, -0.786)$
			$\hat{\mathbf{b}}_1 = (0.076, -0.003, 0.997)$
			$\Delta_b = 2.5°$
$\theta_{y,1}$ (°)	0	-0.2(3)	
λ_2 (nm)	637(2)	635.0(3)	Constrained to [635,639]
$\theta_{x,2}$ (°)	40.5	41.7(3)	$\hat{\mathbf{a}}_2 = (0.665, -0.003, -0.747)$
$\theta_{y,2}$ (°)	0	-0.2(3)	$\hat{\mathbf{b}}_2 = (0.077, -0.003, 0.997)$
			$\Delta_b = 3.2°$
$\theta_{x,p}$ (°)	3.4	6.7(8)	Lens optical axis orientation
$\theta_{y,p}$ (°)	1.6	1.5(7)	$\hat{\mathbf{p}} = (0.116, 0.026, 0.993)$
v_x (pix)	1093	1773(220)	Camera affine transformation x-offset
v_y (pix)	501	998(160)	Camera affine transformation y-offset
θ_{cam} (°)	0	-0.18(2)	Camera affine transformation rotation
f_l/d_{xy}	17 064	17 101(10)	$f_l = 100\,\text{mm}$, $d_{xy} = 5.86\,\mu\text{m}$
f	1	0.670(1)	Correction factor for DC peak strength
w (µm)	5.4	5.3	Constrained to $w \geq 5.3\,\mu\text{m}$
θ_{rot} (°)	94.25	81(8)	$\hat{\mathbf{n}}_{\text{rot}} = (0.045, 0.987, 0.152)$
ϕ_{rot} (°)	90	87(2)	
γ (°)	-16.955	-17.7(2)	
α	0.001		Relative strength of the position vs. intensity loss

TABLE S2. DMD parameters inferred from validation experiments compared with expected values.

E. DMD Model Validation

We validate our modelling approach by displaying different SIM-like patterns on the DMD and measuring the intensity and position of the resulting diffraction orders on a camera

placed behind a lens in a 2f-configuration with the DMD[3] (Fig. S5A). For each pattern, we determine the position and intensity of the five most prominent diffraction orders by fitting each peak to a 2D Gaussian. We normalize each intensity by the DC peak's intensity. We additionally normalize each peak intensity to the expected diffracted intensity based on the strength of each Fourier mode in the DMD pattern. Then, we compare the resulting normalized intensities and positions to the values predicted by our DMD diffraction model (Fig. S5B). We fit the model using a non-linear least-square approach to determine the beam input angles and wavelengths, the DMD mirror grid and mirror rotation parameters, and the imaging system alignment parameters. To obtain a robust parameter uncertainty estimation, we apply a bootstrapping procedure using 1000 samples[5]. We report the best-fit parameters using the full dataset together with the standard deviation of the parameters as determined using the bootstrap in table S2.

For the fit, we use different parameterizations of the parameters than elsewhere. For example, we describe the input directions in terms of angles θ_x and θ_y, where

$$\hat{\mathbf{a}} = (\tan\theta_x, \tan\theta_y, -1) \,/\sqrt{\tan^2\theta_x + \tan^2\theta_y + 1}.$$

This is convenient, as these two angles are independent and have an unbounded domain, simplifying the optimization compared with working with unit vector components. Also, the parameterization ensures $a_z < 0$, so the represented vector is incident on the DMD. Alternatively, for the rotation axis, we adopt the parameterization

$$\hat{\mathbf{n}}_{\text{rot}} = (\cos\phi_{\text{rot}}\sin\theta_{\text{rot}}, \sin\phi_{\text{rot}}\sin\theta_{\text{rot}}, \cos\theta_{\text{rot}}) \,.$$

This again provides two unbounded independent variables but supports unit vectors with any sign for their z-component.

We find that the inferred DMD model parameters differ somewhat from the nominal values. The rotation axis of the $-$ mirrors is oriented about 13° different than expected, and the mirror rotation angle is $-17.7°$, slightly larger than expected. These parameters imply that the best achievable performance for 2-color operation with this DMD is somewhat worse than predicted for the nominal values (see fig. S5 C). In particular, the predicted blaze angle violations are on the order of approximately 3°, compared with approximately 0.5° as found in Fig. 2E.

S8. SEQUENCE DIAGRAM FOR ACQUISITION SCHEME USING IMSWITCH AND AUXILIARY HARDWARE COMPONENTS

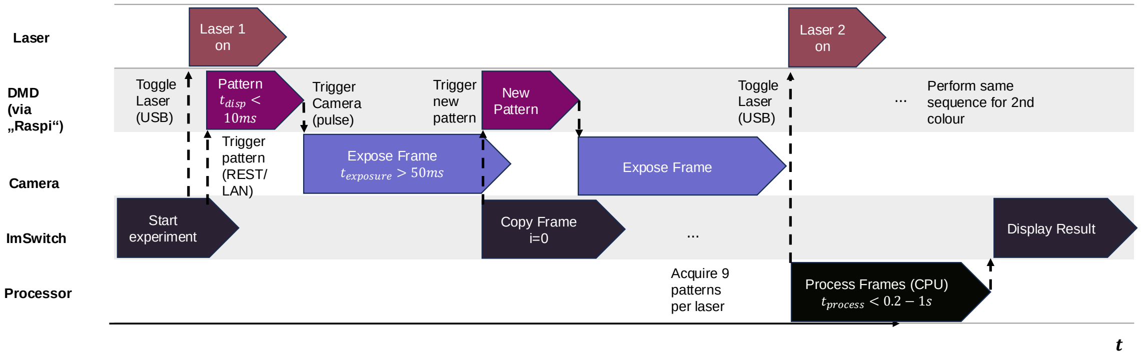

FIG. S6. **Trigger Diagram for the different hardware components:** The trigger diagram illustrates the temporal coordination between various devices and image processing routines. The primary bottleneck in imaging speed is the camera sensor's exposure time, $t_{\text{exposure}} > 50\,\text{ms}$, largely due to the relatively low laser power reaching the sample. To avoid additional delays in the acquisition process, frame acquisition (i.e., transferring data from the camera to random access memory, (RAM)) and image processing are handled asynchronously. The Digital Micromirror Device (DMD), controlled by a Raspberry Pi, serves as the master trigger, dictating the camera's frame acquisition. ImSwitch monitors the availability of the framebuffer and manages system parameters, including toggling the laser, pausing experiments, and handling image stack reconstruction and display.

S9. HARDWARE AND SOFTWARE INTEGRATION USING IMSWITCH

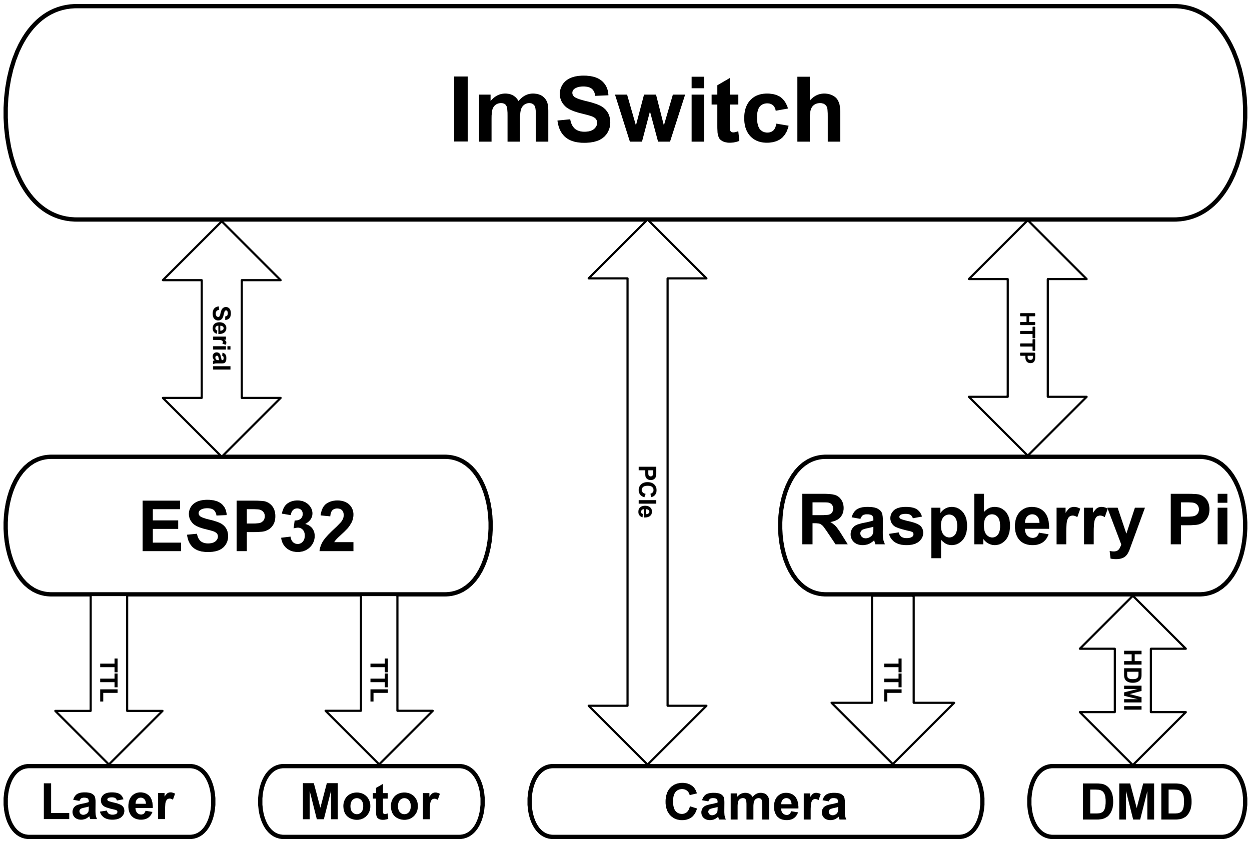

FIG. S7. **Flowchart of the communications channels for image acquisition:** ImSwitch is the hardware orchestration, frame acquisition and image processing software that organizes displaying of the SIM patterns, motor control, camera frame readout and real-time SIM image reconstruction. Device orchestration is facilitated by ImSwitch, with a Raspberry Pi handling rapid pattern display on the DMD via HDMI and triggering the camera, while UC2-REST manages additional hardware controls, such as syncing focus position and laser intensity.

S10. BILL OF MATERIALS

An up-to-date version of this list can be found in the online documentation `https://opensimmo.github.io/docs/02_1_BillOfMaterials`. Design and CAD files required for 3D printing and laser cutting custom parts are available at `https://opensimmo.github.io/docs/02_2_Preparation`. This also involves the creation of the Fourier masks (`https://opensimmo.github.io/docs/02_2_Preparation#fourier-mask`.

ID	Part	Description	Qty	Cost (€)	Link
	Acrylic glass	6mm thickness, 60x60mm, black for enclosure	3	50	Kunststoffplattenonline
DLP4710EVM-G2	DMD	Pattern generation	1	1100	Texas Instruments
AC254-050-A	Achromatic lens	Laser collimation (L1/L2)	2	80	Thorlabs
AC254-075-A	Achromatic lens	Telescope (L3/L4)	2	80	Thorlabs
AC254-200-A	Achromatic lens	Tube lens	1	80	Thorlabs
PF10-03-P01	Silver coated mirror	Laser direction (M1-M3)	3	50	Thorlabs
KM100	Kinematic mirror mount	M1-M3	4	38.04	Thorlabs
FL488-30	Fiber coupled diode laser	488nm 30mW,4 µm core	1	250	openUC2
FL635-60	Fiber coupled diode laser	635nm 60mW,4 µm core	1	250	openUC2
CP33/M	Optomechanical cage plate	Optical parts mount	6	17.35	Thorlabs
SM1FC	Fiber adapter	Fiber adapter (LD1/LD2)	2	30.7	Thorlabs
CXY1A	XY translation mount	Positioning Fourier mask	1	183.14	Thorlabs
	Fourier mask	Aluminum foil pierced with pin	1		custom
SM1L40	Lens Tube	Tube lens mount	1	45.12	Thorlabs
	Raspberry Pi 3B+	4x 1,4 GHz, 1 GB RAM	1	38.65	Reichelt
ESP32-D1-R32	ESP32	Compatible with CNC controllerboard	1	9.99	Amazon
A4988	CNC-Controllerboard	Motor driver	1	17.7	Reichelt
17HS4417P1X4	Nema17 Motor	z direction control	1	15	Reichelt
	GT2 150mm	Focus control belt	1	2.40	I3DService
	Pulley GT2	Stepper motor pulley	1	1.68	I3DService
	HDMI Cable	Display connection	1	2.7	Reichelt
RPI-PS-12.5EU-WT	microUSB powersupply	5.1V/2.5A for Raspberry pi	1	8.4	Reichelt
DIN912	M3 Screw Set	Mechanical parts montage	1	15	Amazon
P200/M	Mounting Post	Enclosure height	3	60.86	Thorlabs
PF125B	Clamping Fork	Post fixation	3	14.68	Thorlabs
C1515/M	Mounting Post Bracket	Enclosure leviation	3	106.91	Thorlabs
DIN EN 573-3	Aluminium Profile	20mm mount for RailOptics	3	7	Profilzuschnitt24

TABLE S3. **Bill of Materials**. Abbreviations in description indicate where part appears in the system diagram, Fig. 1.

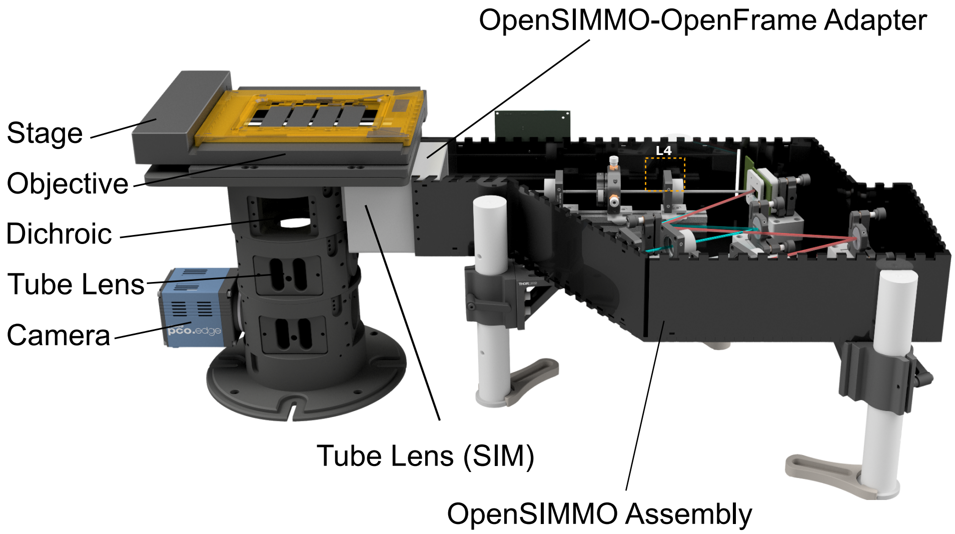

FIG. S8. **Rendering of the openSIMMO attached to another open-source system:** The openSIMMO setup can easily be adapted to other microscope bodies. By teaming up with other open-source projects like OpenFrame, one can build a powerful super-resolution microscope for a modest budget, with full control over the hardware and a reasonable amount of effort to build it. The expertise needed to replicate such a setup no longer requires an engineering degree. The support of the scientific community can help solve problems and improve the design over time. The modular design of the OpenFrame enables the integration of custom modules for e.g. autofocus or other fluorescent techniques.

REFERENCES

[1]H.-W. Lu-Walther, M. Kielhorn, R. Förster, A. Jost, K. Wicker, and R. Heintzmann, "fastSIM: a practical implementation of fast structured illumination microscopy," Methods and Applications in Fluorescence **3**, 014001 (2015).

[2]M. Müller, V. Mönkemöller, S. Hennig, W. Hübner, and T. Huser, "Open-source image reconstruction of super-resolution structured illumination microscopy data in ImageJ," Nature communications **7**, 10980 (2016).

[3]P. T. Brown, R. Kruithoff, G. J. Seedorf, and D. P. Shepherd, "Multicolor structured illumination microscopy and quantitative control of polychromatic light with a digital micromirror device," Biomed. Opt. Express **12**, 3700–3716 (2021).

[4]T. I. Inc., "TI designs: TIDA-01474 low-latency, high-speed TI DLP(R) digital projection reference design," (2017).

[5]B. Efron, "Bootstrap Methods: Another Look at the Jackknife," The Annals of Statistics **7** (1979), 10.1214/aos/1176344552.
